# Efficacy of *Panax ginseng* Meyer Herbal Preparation HRG80 in Preventing and Mitigating Stress-Induced Failure of Cognitive Functions in Healthy Subjects: A Pilot, Randomized, Double-Blind, Placebo-Controlled Crossover Trial

**DOI:** 10.3390/ph13040057

**Published:** 2020-03-29

**Authors:** Pierre-Antoine Mariage, Areg Hovhannisyan, Alexander G. Panossian

**Affiliations:** 1 Botalys SA, 8 Quai des Usines, 7800 Ath, Belgium; pa.mariage@botalys.com; 2 Sport Medicine and Antidoping Service Republican Centre, Yerevan 0001, Armenia; dopingareg@gmail.com; 3 Phytomed AB, Bofinkvagen 1, 31275 Vaxtorp, Sweden

**Keywords:** *Panax ginseng*, hydroponic cultivation, stress, fatigue, cognitive performance

## Abstract

Background: The aim of this pilot study was to compare the efficacy of hydroponically cultivated red *Panax ginseng* Meyer root preparation (HRG80) and traditionally harvested six-year-old white *P. ginseng* standard preparation (PGS) with placebo in preventing symptoms of stress. Methods: The effects of HRG80, PGS, and placebo capsules were studied in 50 tired healthy subjects in a three-arm, randomized, double-blinded, placebo-controlled crossover trial. Efficacy-outcome measures included the accuracy of processing the d2 test for cognitive functions, obtained accuracy score in a computerized memory test, and the perceived-stress (PS) score. Results: A statistically significant interaction effect between time and treatment (*p* < 0.0001) was observed in the attention d2 and memory tests, indicating that HRG80 treatment was more beneficial than that with a placebo. The effects of PGS were better than those of the placebo, but the difference was not statistically significant. There was significant difference between the effects of HRG80 and PGS (*p* < 0.0001) that were observed after single (Day 1) and repeated administrations on Days 5 and 12 of treatment. Conclusion: Overall, HRG80 treatment was significantly superior compared to that with the PGS and placebo regarding attention, memory, and PS scores after single and repeated administrations for 5 and 12 days.

## 1. Introduction

The *Panax ginseng* Meyer root has been traditionally used in China, Korea, and Japan for thousands of years for the treatment of many conditions, including the age-related decline of cognitive function, general weakness, and for enhancing longevity [1,2,3,4,5,6,7,8]. In Europe, most ginseng preparations are used as a general tonic or adaptogen in cases of fatigue, weakness, and decreased mental and physical capacity [3,8,9,10]. According to the Pharmacopoeia of the People’s Republic of China, the actions of red ginseng (hongshen) root and rhizome are used “to greatly tonify the original qi, regain pulse, and secure collapse” [1], which is in line with the adaptogenic concept that uses modern system-biology and network-pharmacology models to understand the fundamentals of traditional medical systems such as “life vital energy”/qi. [9,10,11,12,13,14,15].

The results of many clinical trials suggesting the beneficial effects of ginseng on stress and cognitive functions were critically reviewed in several comprehensive and systematic review articles [3,15,16,17,18,19]. Overall, ginseng is a promising treatment for mental, industrial, and chronic fatigue [20,21,22], as well as for cognitive functions [23,24,25,26,27,28,29,30,31,32]. Ginseng preparations are currently marketed in the European Union as traditional herbal medicinal products according to Directive 2001/83/EC criteria on the basis of their long-standing traditional use as a “tonic” [3], but not as evidence-based herbal medicinal products with well-established use “due to the heterogeneity of studies regarding investigated preparations and study design, deficiencies in methodological quality, and small numbers of study participants” [3]. The heterogeneity of investigational products is due to a difference in chemical composition, which is dependent on environmental factors, such as climate (temperature, light, rain), soil (pH, fertilization, heavy metals), insects, pests, microbiological infection, processing methods, and storage conditions (light, oxygen), and humidity. Consequently, the reproducible clinical efficacy of herbal preparations can only be achieved with standardized high-quality products, cultivated and manufactured according to the Good Agriculture Practices, Foundation for Food Safety Certification (FSSC2 2000) certified process and GMP Guidelines.

Preparation HRG80 was obtained for the first time by the hydroponic cultivation of ginseng by vertical-farming technology according to an FSSCC2 2000 certified standards ensuring the strict control and stability of growing conditions and, therefore, the reproducible chemical composition and high purity of white ginseng, which is converted into red ginseng HRG80 by steam cooking. The ginsenoside profile of this preparation is similar to ginseng cultivated in the field, but the ginsenoside content is two- to threefold higher. Further processing of this white-ginseng preparation into red-ginseng preparation HRG80 results in the conversion of major highly glycosylated ginsenosides into rare ginsenosides Rg6, Rh4, Rg3, Ck, Rk1, and Rh3.

Currently, there is no scientific evidence that HRG80 preparation is effective in preventing and mitigating the stress-induced deterioration of cognitive functions in healthy subjects. Assuming that new clinical evidence is required for the well-established use of white ginseng in preventing and mitigating stress-induced failure of cognitive functions in healthy subjects, the aim of our study was to demonstrate the clinical efficacy of both hydroponically cultivated red-ginseng preparation (HRG80) and traditionally harvested six-year-old white-ginseng standard preparation (PGS) in commercially available dosage forms. The effects of HRG80, PGS Arkopharma, and placebo capsules on preventing symptoms of stress such as fatigue, impaired memory, difficulty in concentration, and attention deficit related to the daily work situation of healthy teleoperators and information-technology (IT) personnel were studied in a three-arm, randomized, double-blinded, placebo-controlled crossover trial.

## 2. Results

### 2.1. Study Participants, Their Disposition, Baseline Variables, and Treatment Compliance

**Figure A1:**
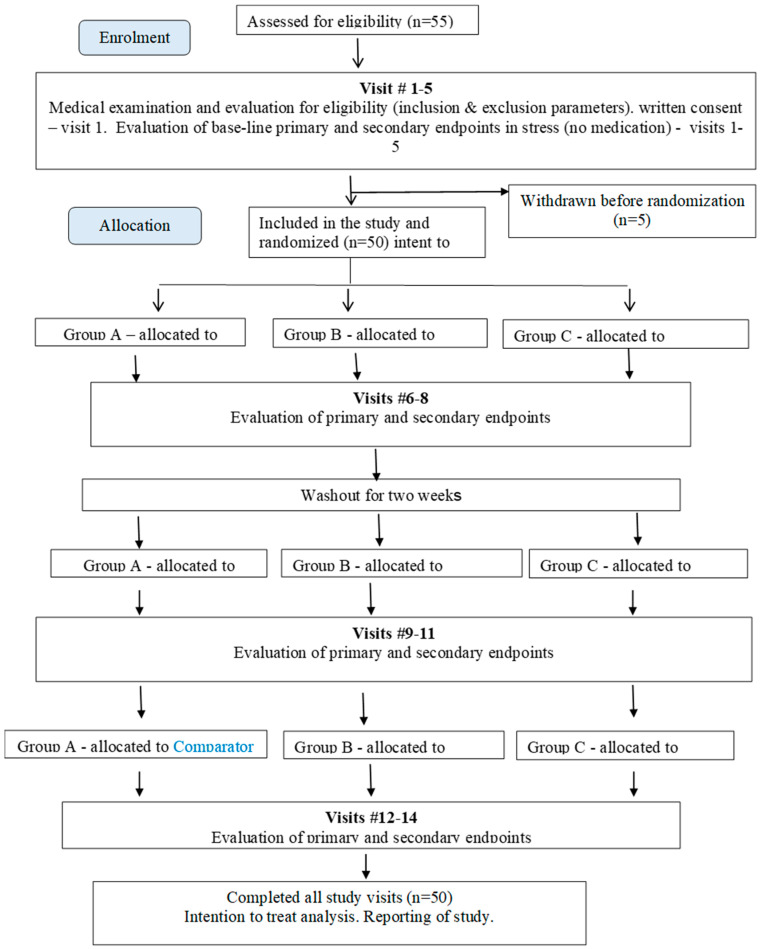
Study design.

Overall, 55 participants were assessed for eligibility; five did not meet the inclusion criteria (Figure A1, Appendix A). Fifty healthy subjects were recruited and included in the study, and all completed treatment. There were no requirements to a specific type of computer work for enrollment in this study. Important was that the participants had a significant mental workload during workdays. All participants who were enrolled and randomly allocated to treatment were included in intention-to-treat (ITT) analysis. Baseline demographic and efficacy-outcome measures are shown in Table 1. The sample size of 50 subjects (Appendix A) included in the study was homogeneous and had normal distribution for age, heart rate, memory, and cognitive-performance scores at baseline, whereas data of the perceived-stress-scale (PSS) test were nonparametric, suggesting nonparametric tools for the assessment of PSS scores. All subjects included in the study were healthy, having a level of occupational stress (PSS = 16.2 ± 2.2) that is over normal limits (13.0 ± 6.2, Appendix C, Box A1), but did not fall into the category of pathological stress. There was no cut-off for PS score at inclusion, and all 50 subjects used all three study preparations at three phases of the trial (Appendix D, Figure A6). There was no statistically significant difference (*p* > 0.8, ANOVA, Kruskal–Wallis test), at the baseline between subgroups (HRG80, n = 17; PGS, n = 16 and placebo, n = 17; *p* > 0.999 Dunn’s multiple-comparison test) entered in the study.

Treatment compliance assessed by a capsule count of the returned preparation comprised 100%; every participant returned 2 of 30 capsules per package, implying that they had consumed 28 capsules during 14 days of treatment (less than nine returned capsules were considered as compliant).

### 2.2. Treatment Efficacy and Safety

#### 2.2.1. Primary Efficacy Endpoint

**Figure 1:**
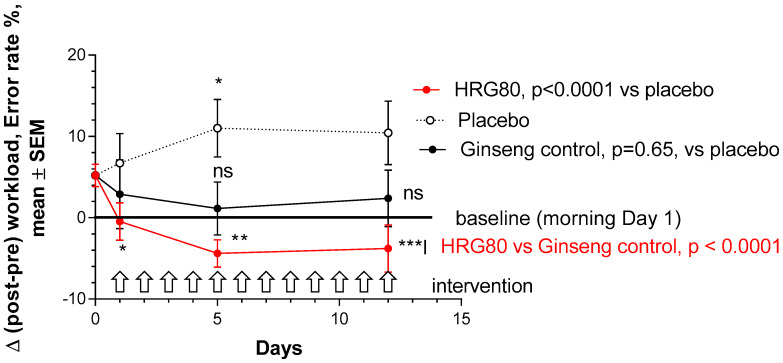
Dynamic changes of post–pre-workload, accuracy score. Changes in attention with time at Days 0, 1, 5, and 12 in placebo, ginseng HRG80, and ginseng control treatment groups. Significance of changes shown by symbols * *p* < 0.05, ** *p* < 0.01, *** *p* < 0.001; ns, not significant. Between-group change comparison over time shows significant difference between ginseng HRG80 and ginseng control treatments (*p* < 0.0001, n = 50 in each group), ginseng HRG80, and placebo treatments, but no significant difference between positive and negative controls.

Error rate (E%) in the attention test was significantly increased at the end of a stressful workday after single and repeated placebo administrations, represented as a change in errors percentage (attention score) from the baseline (morning) (Δ was >0; Figure 1 and Appendix D, Figure A4).

HRG80 significantly decreased Δ (compared to baseline Day 1—no treatment), which was negative during all 12 days of treatment, indicating that mental performance was significantly higher with single and repeated administrations of HRG80. Within-group comparison showed a statistically significant decrease of errors from Day 0, *p* = 0.0047 (Appendix D). The error percentage was still higher (insignificant, *p* > 0.05) in the afternoon test than in the morning tests after both single and repeated administrations of control ginseng (Arkophama) preparation (Appendix D). Within-group time-dependent repeated measures showed no statistically significant decrease of error percentage from Day 0, *p* = 0.7 (Appendix D).

A statistically significant interaction effect between time and treatment (*p* < 0.0001) was observed in the attention d2 test, indicating that HRG80 treatment was more beneficial than that with a placebo (Figure 1).

**Figure 2:**
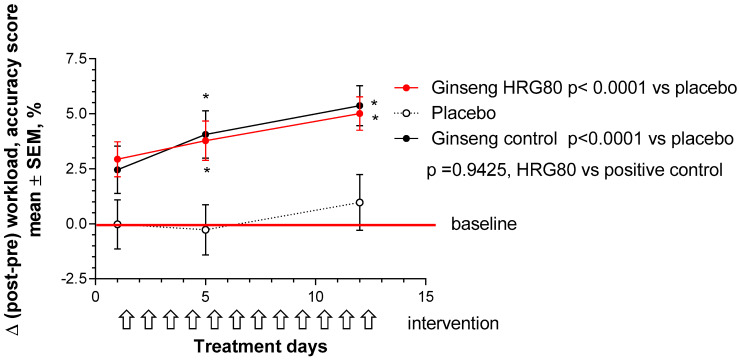
Changes of MT scores from baseline during whole study period; two-way ANOVA. Change in memory with time at Days 0, 1, 5, and 12 in placebo, ginseng HRG80, and ginseng control treatment groups. Significance of changes shown by symbols * *p* < 0.05, ** *p* < 0.01, *** *p* < 0.001l; ns, not significant. Between-group change comparison over time shows significant difference between ginseng HRG80 and placebo treatments (*p* < 0.0001, n = 50 in each group), ginseng control, and placebo treatments, but no significant difference between two ginseng treatments.

There was no significant difference between the effects of ginseng Arkopharma control and placebo (*p* = 0.65). There was significant difference between the effects of ginseng HRG80 and ginseng Arkopharma control (*p* < 0.0001), which was observed after single (Day 1) and repeated administrations on Days 5 and 12 of treatment (Figure 2).

#### 2.2.2. Secondary Efficacy Endpoints

The memory score was nonsignificantly decreased at the end of the stressful workday after single and repeated administrations of the placebo, represented as a change of the accuracy score from baseline (preworkload, morning) to postworkload (Δ < 0, Figure A5 (Appendix D)).

HRG80 significantly increased Δ (compared to baseline day 1—no treatment), which was positive during 12 days of treatment, indicating that mental performance was higher with single and repeated administrations of HRG80. A significant effect was observed on Day 12. Within-group comparison showed statistical significance, *p* < 0.05 (Appendix D). A significant effect of the positive control ginseng (Arkophama) was only observed on Day 12 after repeated administrations. Within-group comparison showed statistical significance, *p* = 0.0005 (Appendix D).

A statistically significant interaction effect between time and treatment (*p* < 0.0001) was observed in the MT, indicating that HRG80 treatment was more beneficial than that with the placebo on Days 5 and 12. The ginseng control treatment effect compared to the placebo effect was also statistically significant (*p* < 0.0001; Figure 2).The difference between HRG80 and ginseng control was statistically nonsignificant (*p* = 0.94; Figure 2).

**Figure 3:**
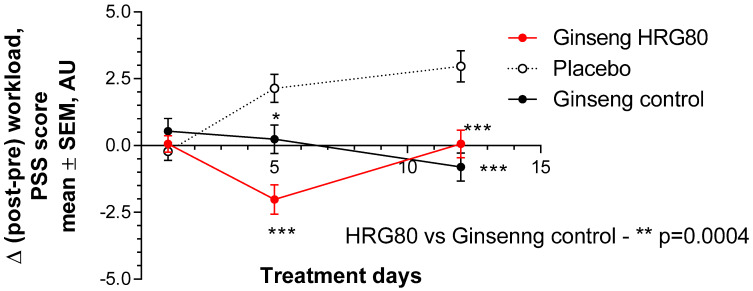
Change of total perceived-stress-scale (PSS) score from baseline during two weeks of treatment. Change in perceived stress with time at Days 0, 1, 5, and 12 in placebo, ginseng HRG80, and ginseng control treatment groups. Significance of changes shown by symbols * *p* < 0.05, ** *p* < 0.01, *** *p* < 0.001; ns, not significant. Between-group change comparison over time showed significant difference between ginseng HRG80 and ginseng control treatments (*p* = 0.0004, n = 50 in each group), ginseng HRG80, and placebo treatments, but no significant difference between positive and negative controls.

The PSS was not significantly changed at the end of the stressful workday after single-dose administration of the placebo, represented as a change from the baseline (pre-workload, morning) to post-workload (Δ was about 0), whereas it was significantly increased after repeated administrations of the placebo on Days 5 and 12 (Figure 3).

Administration of HRG80 significantly decreased Δ (compared to Day 1 of treatment; *p* < 0.0001) after 5 days of treatment, whereas ginseng control decreased PSS after only 12 days of treatment. Within-group comparisons showed a higher effect size and statistical significance for HRG80 treatment (Figure A6; *p* < 0.0001 vs. 0.0236 (Appendix D)).

A very statistically significant interaction effect between time and treatment (*p* = 0.0004) was observed in PSS, indicating that the HRG80 treatment was more beneficial than the ginseng control treatment (Figure 3).

There was a statistically significant difference in PSS score after 5 and 12 days of HRG80 or ginseng control treatment compared to placebo (Figure 3). The change in total PSS score from the baseline was significant even after one week of treatment on Visit 2 in both groups (Figure 3). No significant changes in heart rate were observed from the baseline (morning, before treatment, and stressful workload) to the final workload in healthy subjects during this study.

In total, three troublesome adverse events were observed in 3 of the 50 participants (2% of the sample size, assuming that each participant entered the study three times): diarrhea in one subject taking PGS, and abdominal distention and weariness in two participants taking placebo or HRG80.

No statistically significant difference between HRG80 and placebo was observed regarding toxic adverse events; treatment was well-tolerated, and serious adverse events were not observed.

## 3. Discussion

Ginseng radix and rhizome (*P. ginseng* Meyer) are used in two differently processed forms: air-dried into white ginseng (known in China as renshen), and steamed at 100 °C to red ginseng (hongshen), which contains no less than 0.40% of the sum of ginsenosides Rg1 and Rb1 (dried drug) [1,33]. Consequently, the chemical composition of red and white ginseng differs [4]. Most of the pharmacological actions of ginseng are attributed to one type of tetracyclic triterpenoid glucoside (saponins), namely, the ginsenosides, which have a range of pharmacological activities including immunomodulatory, anticancer [34], antifatigue, antiaging [35], antidiabetic, antidepressant-like [36], and neuroprotective effects [37].

To date, 112 saponins have been reported as components of *P. ginseng*, also known as Korean ginseng; >80 of them are isolated from raw or processed ginseng, and the others are acid/base hydrolysates, semisynthetic saponins, or metabolites [38,39]. A total of 39 ginsenosides were identified and quantified in ginseng roots, leaves, stems, and berries, where the roots’ major ginsenosides are M-Rb1 (0.47%), Rb1 (0.38%), Rf (0.46%), Ra2 (0.32%), Rg1 (0.31%), Re (0.3%), and Rg2 (0.17%), whereas the content of other minor ginsenosides is less than 0.1% [40]. The effects of ginsenosides Rb1, Rg1, Rg2, Rg3, Ro, Rb3, Rd, Rf, Rh2, and 20(S)PT on the nervous system and the behavior of animals were demonstrated in numerous studies [3]. Ginsenosides Rb1, Rd, Re, Rg1, Rg2, Rg3, Rh1, Rh2, Rh3, PF11, and NTR1, gintonin, and compound K showed potential activity in treating cognitive deficits [41].

**Figure A2:**
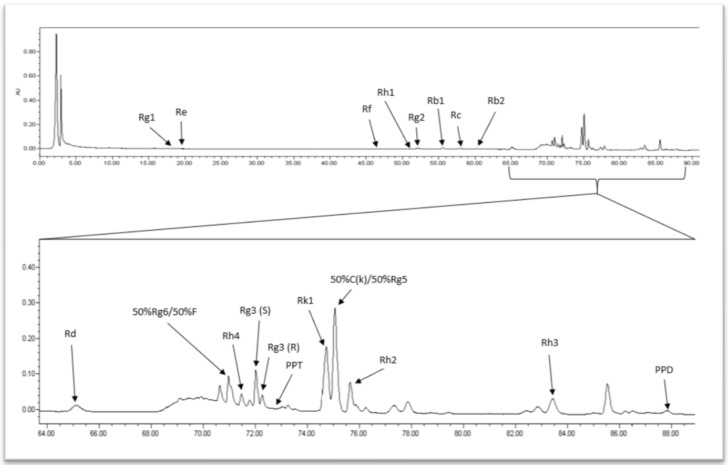
HPLC profile of ginsenosides of HRG80 capsules. Analytical powder sample from 10 capsules was exhaustively extracted by 70% methanol and analyzed by a Waters LC system using reverse-phase HPLC column ACE C18 5 μm (250 × 4.6 mm) and the solvent system containing gradually increasing concentration (from 21% to 90% in eight steps) of acetonitrile in a water solution of 0.1% orthophosphoric acid at 30 °C, flow rate, 1.2 mL/min; diode array detection at 203 nm. The content of analytical markers in the capsules was quantified by reference standards used for the generation of calibration curves. The analytical method was validated for selectivity, precision, and accuracy (RSD < 5%). Reference standards purchased from Sigma-Aldrich (now Merck KGaA, Darmstadt, Germany) and Extrasynthese (Genay, France):

The content of active ginsenosides in wild growing roots is normally in the range of 0.6–3.0%, as determined by the mass of the root powder [33]. The content of active ginsenosides in a representative sample of *P. ginseng* preparation is about 2.6%, as determined by the mass of the root powder (Table 2 and Figure A2 and Table A1 in Appendix B).

Major ginsenosides Rg1, Re, Rb1, Rb2, Rc, Rd, and Rf have poor bioavailability [41,42], but can be metabolized by intestinal microflora (gut bacteria) or gastric juice [41,43,44,45,46,47,48,49,50] into more bioavailable and active [3,38,49,50,51], minor, so-called “rare” ginsenosides Rg2, Rg3, Rg6, Rh2, Rh3, Rh4, compound K, Rk1, and PPD [7,38,39,41,46,52], comprising 0.8% as determined by mass of the root powder (Table 3). The content of more bioavailable rare ginsenosides in the *P. ginseng* HRG80 preparation, obtained by hydroponic cultivation in controlled stressful conditions, followed by traditional cooking of roots, was 7.8-fold higher (Table 3), suggesting the superior pharmacological activity of the HRG80 preparation compared with traditionally harvested six-year-old red *P. ginseng.*

Cultivation of *P. ginseng* in tissue culture, which includes not only tissue (e.g., meristem and leaf tissue) and cell culture, but also organ cultures (e.g., roots, hairy roots), is a promising approach to increase the content of targeted secondary metabolites under controlled conditions [53]. It is presumed that this approach is most suitable and effective for good manufacturing practices [53,54]. In particular, soilless cultivation of ginseng in hydroponics and aeroponics cultures is most appropriate due to many reasons [55,56], including the fact that this method is safe and free of pesticides and agrochemicals [57,58]. Parameters including nutrient-solution composition, pH, aeration, temperature, light, and numerous stressful factors are crucial not only for optimal plant growth and development, but also for the increased biosynthesis of pharmacologically active secondary plant metabolites that play important roles in plant defense response and survival [55,56,59]. Choi et al. [55] cultivated *P. ginseng* in hydroponics and observed higher ginsenoside and phenolic content in its leaves.

The primary objective of the study was to compare the efficacy and tolerability of hydroponically cultivated *P. ginseng* preparation HRG80 with a placebo and an active control white-ginseng preparation (Arkopharma), with respect to the effects on cognitive functions, memory, and stress-induced fatigue in healthy subjects.

Social-stress impact often becomes the trigger for acute mental fatigue and the onset of chronic-fatigue syndrome as a result of the persistent accumulation of acute fatigue [60]. Mental fatigue is associated with decreased attention and reduced ability to concentrate, resulting in failure to complete mental tasks, which could negatively affect work performance [60]. That is of primary importance for many healthy subjects involved in social networks, who are using computers on a regular basis. Therefore, the participants of this study were individuals occupied in social service—teleoperators, engineers, and IT personnel who are permanently overloaded with cognitive tasks and exposed to social stress.

**Figure A3:**
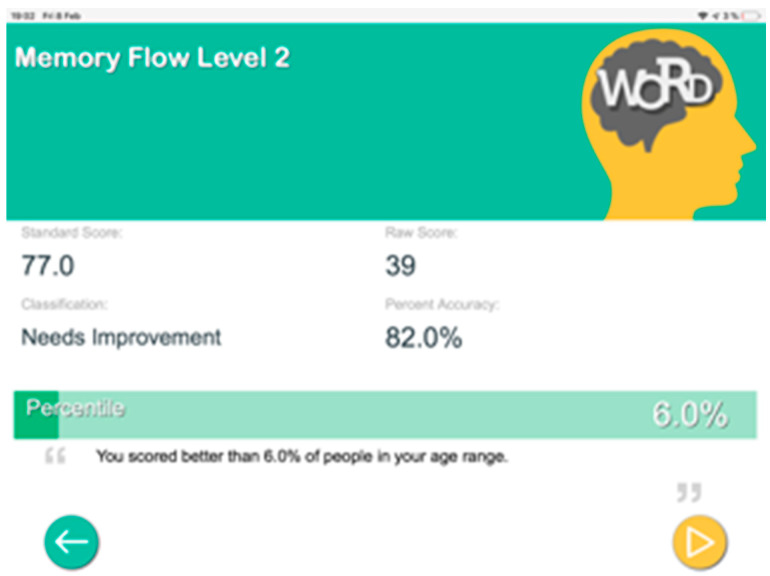
An example of the results of testing

**Figure A4:**
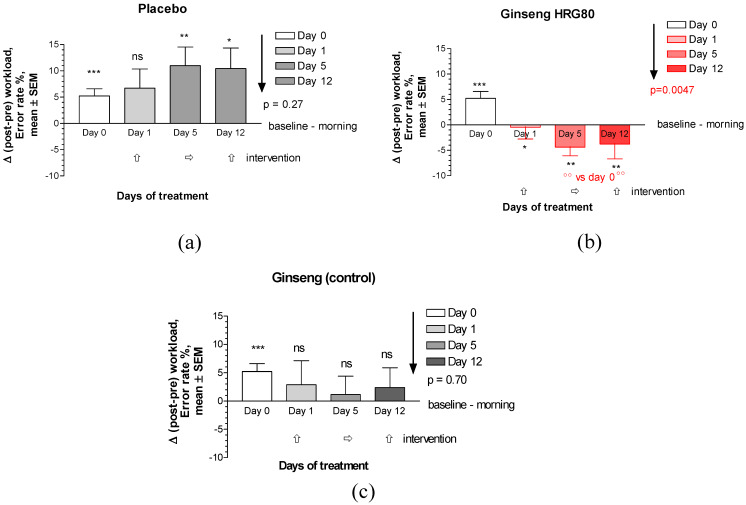
Difference between post- and preworkload attention scores (error %, mean ± SEM, *Y*-axis) in d2 test at Days 0, 1, 5, and 12 in (**a**) placebo, (**b**) ginseng HRG80, and (**c**) ginseng control treatment groups. Change significance shown by symbols * *p* < 0.05, ** *p* < 0.01, *** *p* < 0.001; ns, not significant. Within-group improvement of cognitive function on stress background at end of treatment (Day 12 vs. Day 0) was observed only during the treatment with ginseng HRG80 (*p* = 0.0047).

**Figure A5:**
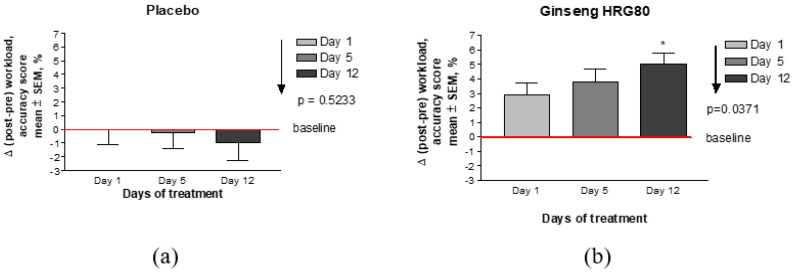

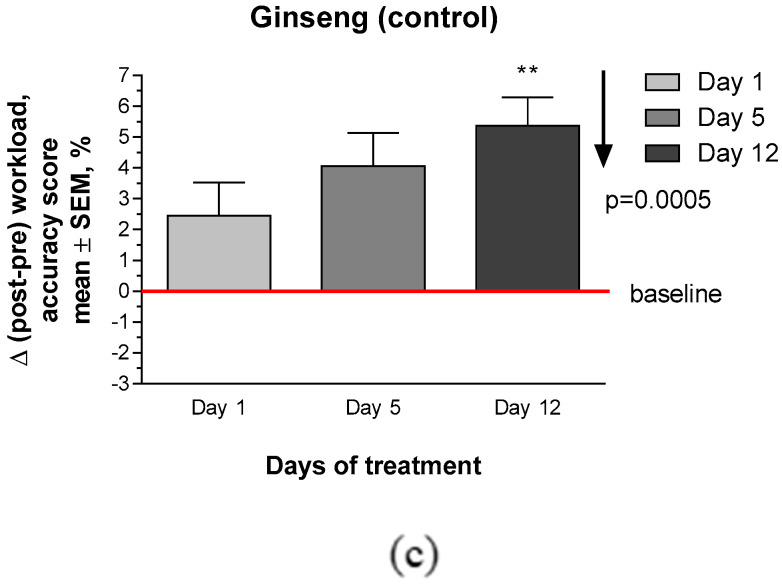
Difference between post- and preworkload accuracy scores (mean ± SEM, *Y*-axis) in memory test at Days 0, 1, 5, and 12 in (**a**) placebo, (**b**) ginseng HRG80, and (**c**) ginseng control treatment groups. Change significance shown by symbols * *p* < 0.05, ** *p* < 0.01, *** *p* < 0.001; ns, not significant. Within-group improvement of mental performance on stress background at end of treatment (Day 12 vs. Day 0) was observed during treatment with ginseng groups (p < 0.05), but not during treatment with placebo (*p* > 0.5).

**Figure A6:**
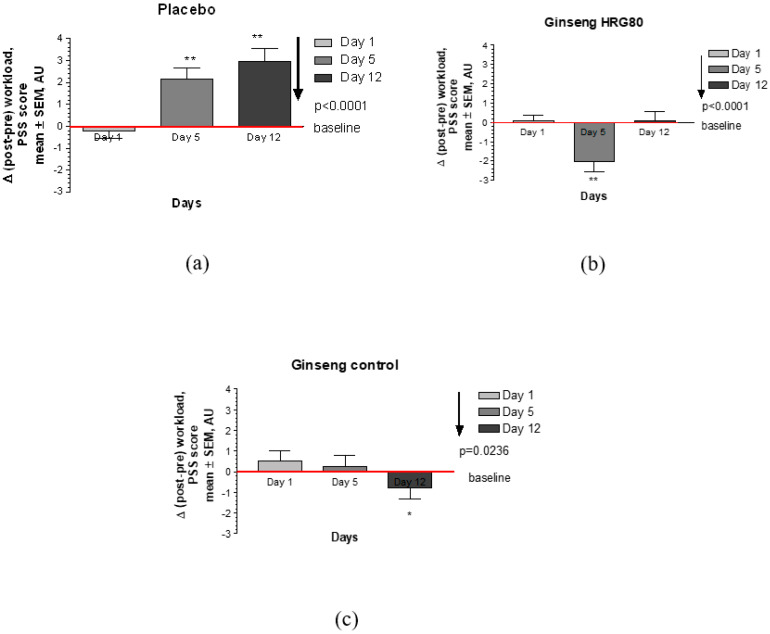
Difference between post- and preperceived stress scores (PSS, mean ± SEM, *Y*-axis) in memory test at Days 0, 1, 5, and 12 in (**a**) placebo, (**b**) ginseng HRG80, and (**c**) ginseng control treatment groups. Change significance shown by symbols * *p* < 0.05, ** *p* < 0.01, *** *p* < 0.001; ns, not significant. Within-group improvement observed in ginseng groups on Day 5 in HRG80 treatment (Day 5 vs. Day 0) and on Day 12 in ginseng control group; in place group, perceived stress increased over time (*p* < 0.001).

Efficacy was studied and evaluated using commonly accepted tests [61,62,63] for perceived stress (PS), attention, concentration, and visual-scanning speed, and a computerized MT for the assessment of cognitive functions (learning, memory, and attention; for details, see Box A1 and Figure A3 in Appendix C).

A statistically significant interaction effect between time and treatment was observed in the attention d2 and memory tests, indicating that HRG80 treatment was more beneficial than that with the placebo. There was no significant difference between the effects of PGS and placebo, even though the effect of PGS was superior. There was significant difference between the effects of HRG80 and PGS, which were observed after single (Day 1) and repeated administrations on Days 5 and 12 of treatment. A statistically significant interaction effect between time and treatment was observed in the PS test, indicating that HRG80 treatment was more beneficial than that with the PGS, but not in the MT, in which the difference between HRG80 and PGS was statistically nonsignificant. The number and types of adverse events were the same in all groups; no serious adverse events were observed.

The results of this study are in line with the results of an ex vivo study in rats where HRG80 was more active than SGP in the hippocampal long-term-potentiation (LTP) model; administration of HRG80 and SGP induced higher excitability of hippocampal pyramidal cells in vitro, leading to LTP compared to the placebo [64].

A randomized, double blind, placebo-controlled study to determine if red-ginseng preparation HRG80 is superior to conventionally grown ginseng and a placebo on electrical brain activity in elderly subjects during performance of various cognitive tasks is now in progress [65].

Overall, further confirmatory studies with a higher number of patients and a longer treatment period are needed to provide more evidence for the superiority of red ginseng versus white ginseng and a placebo.

Results of the clinical efficacy of various ginseng preparations in mental and chronic fatigue are conflicting. Thus, Hallstrom et al. (1982) studied the effects of radix ginseng in fatigued night nurses and concluded that ginseng exhibits antifatigue activity in a placebo-controlled, double-blind crossover trial [20]. However, there were no significant differences in the computerized neurocognitive-function test system scores between the red-ginseng group and the placebo group in a two-week pilot study in 20 healthy young males with no psychiatric or cognitive problems [27]. Results on a larger group of 90 healthy volunteers with mild cognitive impairment suggested that Korean ginseng has a cognition-enhancing effect [29].

Ten trials with good methodology (Jadad score of more than 3 points in five of the eight trials) evaluated the efficacy of ginseng on psychomotor function using white or red ginseng [3,24,30,31]. The studies yielded six positive and two negative findings. Authors reported the anti-mental-fatigue effects of *P. ginseng* extract (G115) by showing improvements in the cognitive performance and memory of healthy volunteers in serial clinical studies [23,24,25,26]. However, no significant difference was found between ginseng and the placebo [18,25].

In this context, the results of our study suggesting that ginseng HRG80 is a promising treatment for fatigue are in line with the results of clinical studies of other adaptogenic plant extracts that exhibit antifatigue effects increasing the mental performance and cognitive functions of healthy subjects and patients with chronic fatigue [66]. In contrast to conventional stimulants (e.g., caffeine), sympathomimetics (ephedrine), and tonics such as cola [67], adaptogens have no adverse events (headache, fatigue, etc.) associated with withdrawal [68,69].

## 4. Materials and Methods

### 4.1. Participant Eligibility

A study of the efficacy of the proprietary *P. ginseng* HRG80 preparation in healthy subjects with symptoms of stress and fatigue was conducted in the Sports Medicine and Antidoping Service Republican Center (SMADS, Yerevan, Armenia) with approval of their Ethics Committee (approval date: 22 February 2017). The study was performed in accordance with the Declaration of Helsinki (52nd WMA General Assembly, Edinburgh, Scotland, October 2000).

This trial was registered at ClinicalTrials.gov (identifier: NCT03947554; https://clinicaltrials.gov/ct2/show/NCT03947554?term=ep-1005&rank=1). All participants provided written informed consent to participate in the study prior to being screened.

### 4.2. Study-Population Selection

Fifty-five healthy volunteers of both sexes, aged 18 to 65 years old, were assessed for eligibility; fifty subjects were enrolled in the study and included in intention-to-treat (ITT) analysis. Participants were eligible for participation in this trial in the period from April to June 2019. Individuals were recruited by doctors from SMADC, and they included the teleoperators, engineers, and IT personnel of VivaCell-MTS Ltd. (Yerevan, Armenia). All subjects included in the study were healthy and had almost the same level of occupational stress, which does not fall into the category of pathological stress. There was not a cut-off for PS score at inclusion. This subjective self-assessment score at the baseline did not have Gaussian distribution, but the sample size of 50 subjects was homogeneous before treatments, with standard deviation of 2.24 at a mean value of 16.2, and 13.8% coefficient of variation that was suitable for the nonparametric statistical assessment of PSS used in this study.

#### 4.2.1. Recruitment and Screening

During the initial visit of the study site, SMADC, inclusion and exclusion criteria were evaluated, and individuals interested in study participation received information about the study. If individuals were suited for study participation, an appointment for medical screening, including anamnestic evaluation, was made. Subjects who were temporarily taking over-the-counter symptomatic medications or dietary supplements that may have potential effects on cognitive functions were told to stop that treatment at least 2 weeks before beginning the study (wash-out period). They were also informed to not consume more than one cup of coffee daily (in the morning) during the study (1 week).

#### 4.2.2. Inclusion Criteria

The target population comprised subjects experiencing stress and fatigue during 8 h of working at the computer, but who were otherwise healthy. The inclusion criteria were as follows: men and women 18–65 years of age (any race and ethnicity) who could understand and provide signed informed consent and were able to participate in an 11-week study.

#### 4.2.3. Exclusion Criteria

The exclusion criteria were as follows: taking over-the-counter medications or dietary supplements that may have potential effects on cognitive functions; consuming more than one cup of coffee daily (in the morning); allergy to ginseng preparations; and any other condition that precluded participation according to the judgement of the investigator.

#### 4.2.4. Participant Withdrawal

Participants were free to withdraw from the study at any time without giving a reason and with no negative consequences. There were no cases of participant withdrawal from the study.

### 4.3. Study Design

This was a randomized, double-blind, placebo-controlled, three-arm parallel-group crossover trial of the efficacy of hydroponically cultivated *P. ginseng* Meyer root preparation (HRG80) compared to traditionally harvested 6-year-old red PGS preparation Arkopharma (positive control) and a placebo (negative control) in preventing symptoms of stress such as fatigue, impaired memory, difficulty in concentration, and attention deficit related to the daily work situation of healthy subjects. The effects of HRG80, PGS Arkopharma, and placebo capsules, taken for 14 days, were studied (diagram is shown in Figure A1, Appendix A).

### 4.4. Intervention and Comparator

The herbal-medicine intervention used in this trial was an HRG80 420 mg capsule containing 210 mg powdered herbal preparation of red ginseng root HRG80 (lot #PGS190114-001, Botalis SA, Ath, Belgium). The powdered herbal preparation was obtained from hydroponically cultivated Korean ginseng (*P. ginseng* Meyer) root in controlled conditions to contain 12%–15% total ginsenosides and 10%–12% rare ginsenosides. Harvested ginseng roots were air-dried and steamed at 130 °C for 2 h to obtain red ginseng roots. Then, the red ginseng was powdered and sifted at 300 µm, and standardized for rare-ginsenoside content and ginsenoside profile. One HRG80 capsule (418 mg), manufactured by Botalys SA, was standardized for the content of 209 mg powdered *P. ginseng* Meyer root powder corresponding to 31.8 mg of ginsenosides Rg1, Re, Rf, Rh1, Rg2, Rb1, Rc, Rb2, Rd, Rg6, Rh4, Rg3, PPT, Rk1, C(K), Rh2, Rh3, and PPD; 25.9 mg of rare ginsenosides Rh1, Rg2, Rg6, Rh4, Rg3, PPT, Rk1, C(K), Rh2, Rh3, and PPD; and 209 mg of inactive excipient rice flour. The comparator was *P. ginseng* Arkopharma capsules; one capsule contained 382 mg powdered *P. ginseng* Meyer root corresponding to 9.8 mg of total ginsenosides Rg1, Re, Rf, Rh1, Rg2, Rb1, Rc, Rb2, Rd, Rg6, Rh4, Rg3, PPT, Rk1, C(K), Rh2, Rh3, and PPD; and 3.056 mg of rare ginsenosides Rh1, Rg2, Rg6, Rh4, Rg3, PPT, Rk1, C(K), Rh2, Rh3, and PPD, calculated as ginsenoside Rb1. The placebo capsule contained 418 mg rice flour and brown sugar. Herbal-preparation quality was tested by HPLC (B) in accordance with specifications using appropriate reference standards. All analytical methods were validated for selectivity, accuracy, and precision. Study preparations were packed and labeled by Botalys SA as per national requirements regarding their use for clinical-trial investigations. The label included the drug name, study code, and storage conditions. Reference samples were retained and stored at Botalys SA.

#### 4.4.1. Doses and Treatment Regimens

Participants received a labeled paper box containing either: HRG80, 418 mg capsules (containing 31.7 mg ginsenosides corresponding to 209 mg powdered ginseng roots); PGS Arkopharma, 384 mg capsules (containing 9.9 mg ginsenosides corresponding to 384 mg *P. ginseng* root); or placebo, 400 mg capsules, containing brown sugar and rice flour that had to be taken orally.

The participants were instructed to take two capsules once a day with water for 2 weeks.

The appearance, taste, smell, and color of all three preparations were similar and organoleptically undistinguishable. The PI was responsible for maintaining accountability records for the study products.

#### 4.4.2. Randomization and Blinding

Study preparations were randomly labeled by a qualified pharmacist (QP, not involved in the study) at the manufacturing site in accordance with a random sequence generated using the random-number generator in GraphPad software (2017 online version; https://www.graphpad.com/quickcalcs/randomize1.cfm). It contained information about the content of each package, namely, how the placebo, HRG80, and PGS Arkopharma containers were encoded. The GraphPad-generated table contained four columns and 51 lines; the left column had the subjects’ numbers from 1 to 50; the other columns showed the assigned treatments (A, B, and C) that were taken during Phases 1, 2, and 3. These three columns were filled with randomly distributed unique letters A, B, and C (treatment code). The treatment code showed that HRG80 was assigned as Treatment A, placebo as Treatment B, and the comparator Arkopharma capsules as Treatment C.

#### 4.4.3. Allocation Concealment

The random sequence of treatment codes was retained by the QP at the investigational product manufacturing site (sponsor) until the study was completed. It was provided to the PI before statistical evaluation of the study results, when all patients had completed treatment.

#### 4.4.4. Implementation and Blinding

At the first visit, all participants received a consecutive number starting from 1 to 50. Participants were sequentially enrolled by the PI. Each participant was assigned a random treatment number and received the treatment in the corresponding package (treatment code No.). Participants’ allocation sequences, the participant list, identifying the subjects and the study-supplement packages (treatment code No.) were generated and maintained by the PI, who assigned each patient to a treatment code No. (from 1 to 50) and filled the patient name in the case report form (CRF) and on the label of the package. The participant list was used for statistical analysis at the end of the study with the random sequence received from the QP. The treatment code providing information about the actual assignments of the placebo, HRG80, and PGS Arkopharma containers was revealed by the QP after statistical analysis of the results of the study was completed, and the obtained data from the treatment groups were compared. Blinding of trial subjects was performed by using labeled packages containing jars with capsules of identical appearance. Study medication was delivered to the study site prelabeled and coded according to the random sequence. The randomization code was kept secret from the investigators, and the code was only revealed after termination of the study. In this way, the investigators were blinded to the study medication and placebo control, thus ensuring a double-blind design. Information concerning the allocation of participants was kept in sequentially numbered and sealed envelopes that were stored by the chief executive officer (CEO) of the contract research organization. Individual treatment codes, indicating treatment randomization for each randomized participant, were available to the investigator and the sponsor in sealed envelopes. However, there were no break cases from the randomization codes.

#### 4.4.5. Evaluation of Compliance

Participants were asked to take a daily dose of two capsules. Participants were questioned about their overall compliance with the study protocol upon their visits, and the remaining capsules were counted by the study personnel. Compliance was monitored by the PI of the study, who checked the participants’ records in a special form that was attached to the case report form (CRF). The study monitor checked overall compliance with the study protocol upon their visits, and the remaining capsules were counted at the end of the study. All unused capsules (two capsules in each package) were retained in the archive storage of the study site in Armenia.

### 4.5. Study Procedures and Follow-Up

The overview of all visits is presented in Table 3 and Figure A1, Appendix A.

#### 4.5.1. Phase 0, Screening, and Training Visits 1–5

On Visit 1, the screening visit, participants were informed about the details of the pending study, provided with the study medication, and accordingly recruited. All volunteers were checked for eligibility and signed written informed consent, including obligations that they would not take medicine or dietary supplements that may have potential effects on cognitive functions and would not consume more than one cup of coffee daily (in the morning) during the course of the study (1 week). During the first visit, the participants took all tests for cognitive functions and stress twice: in the morning (09:00–10:00, baseline) and in the evening (16:00–18:00). On Visits 2–5 (no treatment), participants took tests for cognitive functions in the morning (09:00–10:00, baseline) and at the end of the workday (16:00–18:00, stress).

#### 4.5.2. Phase I, Treatment, and Assessment Visits 6–8

On Visit 6 (morning, no treatment), participants took all tests for cognitive functions and stress in the morning before starting work (09:00–10:00, baseline). Then, the PI randomly assigned participants to the treatments and gave them the test compound. Participants orally took the Phase I treatment and repeated all tests at 16:00–18:00, at the end of the workday. The treatment lasted 14 consecutive days between Days 8 and 21 of the study. Participants took two capsules per day (two in the morning at 10:00). They also took all tests for cognitive functions and stress on Visit 7 (Day 12) and Visit 8 (Day 21) in the morning (09:00–10:00) and at the end of the workday (16:00–18:00, stress). After the Phase I treatment, participants had a washout period of 14 days (Days 24–37 of the study).

#### 4.5.3. Phase II, Treatment, and Assessment Visits 9–11

On Visit 9 (morning, no treatment), participants took all tests for cognitive functions and stress in the morning before starting work (09:00–10:00). Then, participants orally took Phase II treatment and repeated all tests at 16:00–18:00, at the end of the workday. Treatment lasted 14 consecutive days between Days 38 and 49 of the study. Participants took two capsules per day (two in the morning at 10:00). They also took all tests for cognitive functions and stress on Visits 10 (Day 42) and 11 (Day 49) in the morning (09:00–10:00) and at the end of workday (16:00–18:00, stress). After Phase II treatment, participants had a washout period of 2 weeks (Days 52–65 of the study).

#### 4.5.4. Phase III, Treatment, and Assessment Visits 12–14

On Visit 12 (no treatment), participants took all tests for cognitive functions and stress the morning before starting work (09:00–10:00). Then, they orally took the Phase III treatment and repeated all tests at 16:00–18:00, after the workday. Treatment lasted 14 consecutive days, between Days 66 and 77 of the study. Participants took two capsules per day (two in the morning at 10:00). They also took all tests for cognitive functions and stress on Visits 13 (Day 70) and 14 (Day 77) in the morning (09:00–10:00) and at the end of the workday (16:00–18:00, stress). Then, the study was completed.

### 4.6. Efficacy and Safety Evaluation

The methods of efficacy and safety assessment are described in detail in Appendix C.

#### 4.6.1. Primary Efficacy Outcome

The primary efficacy outcome measure was the processing accuracy of the d2 attention test (d2 test) for attention, concentration, and visual-scanning speed, which was expressed as an error-rate (ER) score (E%), defined as the ratio of errors made to the total number of correct responses during 4 min and 40 s.

#### 4.6.2. Primary Efficacy Endpoint

The primary endpoints were:

- change between baseline (morning, before treatment, and stressful workload) and final workload, as presented by the error-rate score, Δ (post–pre) workload;
- change between baseline and 1, 5, and 12 days of treatment, as presented by Δ error rate score; and
- differences between HRG80, positive control, and placebo at 1, 5, and 12 days of treatment in error-rate score change from baseline, Δ (post–pre) workload.

#### 4.6.3. Secondary Efficacy Outcomes

Secondary efficacy outcome measures were:

- accuracy score obtained by a computerized memory test (MT) for assessment of cognitive functions (learning, memory, and attention), which is expressed as the number of correct responses during 2 min expressed in % (a scale from 0 to 100%); and
- perceived-stress (PS) score that is the only empirically established index of general stress appraisal.

#### 4.6.4. Secondary Efficacy Endpoints

Secondary endpoints were:

- change between baseline (morning, before treatment, and stressful workload) and final workload in PS and MT scores, Δ (post–pre) workload;
- change between baseline and 1, 5, and 12 days of treatment in Δ PS and MT scores; and
- differences between HRG80, positive control, and placebo at 1, 5, and 12 days of treatment in PS and MT scores changes from baseline.

#### 4.6.5. Other Endpoints

Other endpoints were:

- change between baseline (morning, before treatment, and stressful workload) and final workload in heart rate;
- change between baseline and 1, 5, and 12 days of treatment in heart rate; and
- differences between HRG80, positive control, and placebo at 1, 5, and 12 days of treatment in heart-rate changes from baseline.

#### 4.6.6. Safety Outcomes

Safety outcome measures included incidence and severity of adverse events; serious adverse events were assessed by their nature and incidence according to Common Toxicity Criteria, version 4.0.

### 4.7. Statistical Analysis

The clinical data from each visit were recorded using a standardized clinician assessment form. Edited and corrected data were added to an Excel database that was used as input to GraphPad statistical software (San Diego, CA, USA) and Prism software (version 3.03 for Windows). Statistical analysis involved evaluating a patient’s change in scores from the initial visit (baseline) to the intermediate and final visit, and at each scheduled visit of the study. Analysis was performed using “observed” data. The primary population was the ITT population, which was defined as all randomly assigned participants who received at least one dose of study medication. Statistical analysis was performed on an ITT basis for time-to-event endpoints. The alpha level was *p* < 0.05, and the level for tendency was *p* < 0.10. No corrections were made for mass significance. Assessment of baseline characteristics between two groups was done using the Mann–Whitney nonparametric rank-order test or parametric independent-measures t-test. Change significance from baseline at the same time point was tested using the one-sample t-test for parametric variables or the Wilcoxon signed-rank test for nonparametric variables. Analysis of change within treatment groups during the study course was performed with the Friedman nonparametric rank test for repeated measures (4 repeated conditions) in participants who completed all of the tests. The Kruskal–Wallis nonparametric one-way analysis-of-variance (ANOVA) rank-order test, with post hoc Dunn’s multiple-comparison test, was performed on the ITT population in participants who had received at least one dose of study medication. Assessment of the efficacy of the study medications was achieved by comparing the mean changes from the baseline (differences before and after the treatment of every patient) using the Mann–Whitney nonparametric rank-order test or two-way ANOVA, in which an interaction effect indicated a different response over time between the two groups, and would therefore signal a treatment effect.

#### Sample-Size Considerations

We enrolled 50 patients (50 per treatment condition). This was a preliminary study to obtain estimates of parameters that were used to assess and justify the sample-size requirements for a fully powered study. We identified trends in the data that assisted us in refining or modifying the aims of a subsequent proposal. The proposed sample size (n = 100, or 50 per treatment condition) had 90% power to detect an effect size of 0.74 and 80% power to detect an effect size of 0.85 using a two-group t-test with a 0.05 two-sided significance level. Effect size was calculated as the absolute difference in means between the two groups being compared, divided by their common standard deviation (e.g., an effect size of 0.74 corresponded to a difference in mean changes in a d2 test score of 0.74 if the common standard deviation of change was 1.0). We anticipated that we may not have been able to observe a statistically significant difference between treatment conditions in this study, as the observed effect sizes in some recent studies of pharmaceutical agents have been smaller. However, although we anticipated that we could observe an effect size smaller than 0.74 (so that statistical significance was not achieved), we expected to observe nonsignificant trends between treatment conditions, allowing the estimated effect sizes from this project to be used to power a large-scale study of ginseng that is planned as a follow-up to this exploratory study.

## 5. Conclusions

Overall, HRG80 treatment was significantly superior to the positive control and placebo regarding attention, memory, and PS scores after single and repeated administrations at 5 and 12 days. It also had a good tolerability profile.

## Figures and Tables

**Table 1:** Baseline demographic and efficacy-outcome measures. Note: CP, concentration performance; MT, memory test; PS, perceived stress; ns, not significant.

	Age	Heart Rate	CP Score	MT Score	PS Score
**Number of subjects**	50	50	50	50	50
**Mean**	**37.46**	**56**	**25.62**	**88.58**	**16.2**
Std. deviation	8.016	64.50	17.34	6.419	2.241
Std. error	1.134	68.00	2.453	0.9078	0.3169
Lower 95% CI	35.18	66.95	20.69	86.76	15.56
Upper 95% CI	39.74	70.13	30.55	90.4	16.84
**Normality Test**					
KS distance	0.0873	0.08834	0.1734	0.1149	0.2156
*p*-value	>0.10	>0.10	0.0989	>0.10	0.0192
Passed normality test (* = 0.05)?	Yes	Yes	Yes	Yes	No
*p*-value summary	ns	ns	ns	ns	*
Coefficient of variation	21.40%	8.17%	67.69%	7.25%	13.83%

**Table 2:** Ginsenoside content (mg/g) in HRG80, Arkopharma, and placebo capsule powders.

	Ginsenosides	HRG80 (mg/g *)	Arkopharma (mg/g)	Placebo
Major ginsenosides	Rg1	1.26	2.97	0
	Re	1.40	2.77	0
	Rb1	2.73	3.62	0
	Rc	2.32	3.68	0
	Rb2	1.56	3.26	0
	Rd	4.53	0.99	0
	Rf	0.00	0.55	0
Rare ginsenosides	Rg2	1.83	0.23	0
	Rg6	5.04	0.23	0
	Rh4	9.60	1.97	0
	Rg3	8.43	2.22	0
	Rk1	9.97	0.04	0
	C(k)	17.59	0.23	0
	Rh2	2.56	2.19	0
	Rh3	6.04	0.39	0
	PPD	1.11	0.50	0
	**Total**	**76.0 = 7.6%**	**25.8 = 2.6%**	**0**
	Top 7	13.8	17.8	0
	Rare	62.2 = 6.2%	8.0 = 0.8%	0
**Proportion**	**Rare: Major**	**82:18**	**31:69**	**0**

* including excipients −50%.

**Table 3:** Overall study design—healthy patient 14-day treatment, 4-week observation.

	Visit 1 Phase 0 No Treatment	Visits 2–5 No Treatment	Visits 6–8 Phase I Treatment Days 1–14	Washout Period 12 Days	Visits 9–11 Phase II Treatment Days 1–14	Washout Period 12 Days	Visits 12–14 Phase III Treatment Days 1–14
Information	+						
Informed Consent	+						
Clinical Examination	+						
Enrollment and Allocation to IP	+		+ Visit 6				
Test days	1	2–5	8, 12, 21	24–37	38, 42, 49	52–65	66, 70, 77
PSSAQ score	+ *		+ *		+ *		+ *
MAT Tests scores	+ *	+ *	+ *		+ *		+ *
d2 test of attention	+ *	+ *	+ *		+ *		+ *
Hearth rate			+ *		+ *		+ *
Treatment			+		+		+
IP accountability			+		+		+
Adverse Events			+		+		+

* Twice: in the morning (09:00–10:00, baseline) and afternoon (16:00–18:00). PSSAQ: Scores around 13 are considered average. Scores of 20 or higher are considered high stress.

## Appendix B. Identity and Quantity Testing of Active Markers

- ginsenoside Rg1 (purity ≥98%, Extrasynthese, ref. 0101S), retention time 18.4 min;
- ginsenoside Re (purity ≥98%, Extrasynthese, ref. 0103S), retention time 19.5 min;
- ginsenoside Rf (purity ≥98%, Extrasynthese, ref. 0107S), retention time 46.6 min;
- ginsenoside Rh1 (purity ≥90%, Sigma-Aldrich, ref. 56805), retention time 51.3 min;
- ginsenoside Rg2 (purity ≥94%, Sigma-Aldrich, ref. 08171), retention time 52.1 min;
- ginsenoside Rb1 (purity ≥98%, Extrasynthese, ref. 0105S), retention time 55.5 min;
- ginsenoside Rc (purity ≥98%, Extrasynthese, ref. 0106S), retention time 58.0 min;
- ginsenoside Rb2 (purity ≥90%, Extrasynthese, ref. 0104S), retention time 60.5 min;
- ginsenoside Rd (purity ≥98%, Extrasynthese, ref. 0102S), retention time 65.5 min;
- ginsenoside Rg6 (purity ≥90%, Sigma-Aldrich, ref. 43019), retention time 71.1 min;
- ginsenoside Rh4 (purity ≥90%, Sigma-Aldrich), retention time 71.5 min;
- ginsenosides Rg3 (S/R) (purity ≥98%, Sigma-Aldrich, ref. SML0184), retention time 72.0–72.3 min;
- ginsenoside PPT (purity ≥98%, Extrasynthese, ref. 2307), retention time 72.7 min;
- ginsenoside Rk1 (purity ≥95%, Sigma-Aldrich, ref. 42754), retention time 74.7 min;
- ginsenoside CK (purity ≥95%, Sigma-Aldrich, ref. PHL80461), retention time 75.1 min;
- ginsenoside Rg5 (purity ≥90%, Sigma-Aldrich, ref. 43016), retention time 75.1 min;
- ginsenoside Rh2 (purity ≥97%, Sigma-Aldrich, ref. 73658), retention time 75.9 min;
- ginsenoside Rh3 (purity ≥90%, Sigma-Aldrich, ref. 43084), retention time 83.5 min; and
- ginsenoside PPD (purity ≥97%, Sigma-Aldrich, ref. P0031), retention time 87.9 min).

The content of ginsenosides per “HRG80” (418 mg capsule, 6.2% of rare and 7.6% of total ginsenosides), Arkopharma (382 mg capsule, 0.8% of rare and 2.6% of total ginsenosides), and placebo capsules was 15.88, 9.85, and 0 mg/caps, respectively (Table 2 in the main text). Rare ginsenosides were 13 (HRG80) and 3 (Arkopharma) mg/caps.

The purity of the reference standard was analyzed and certified by *Panax ginseng* extract manufacturer Botalys SA.

### HRG80 Purity

As it is grown in a clean, sterile environment, HRG80 is pesticide-, mycotoxin-, irradiation-, solvent-, enzyme-, and GMO-free. Product specification is shown in Table A1.

**Table A1:** Specification of red ginseng, HRG80 capsules.

Parameters	Specification
Loss on drying (IR balance)	<10%
Granulometry	<300 µm
Mycotoxin (external lab)	0 (aseptic culture)
Pesticide (external lab)	0 (aseptic culture)
Ginsenoside content *	Typical value 12–15%
Rare-ginsenoside content **	Typical value 10–12%
DNA identification test (PCR)	Panax ginseng
Heavy metal (external lab)	<10 ppm
Al (external lab)	<5 ppm
Pb (external lab)	<1 ppm
As (external lab)	<0.2 ppm
Cd (external lab)	<0.1 ppm
Hg (external lab)	<0.1 ppm

* Total ginsenosides: Rb1, Rb2, Rc, Rd, Re, Rg1, Rf, compound K, protopanaxadiol, protopanaxatriol, Rg2, Rg3, Rg5, Rg6, Rh1, Rh2, Rh3, Rh4, Rk1 active substance (powdered roots). ** Rare ginsenosides: compound-K, protopanaxadiol, protopanaxatriol, Rg2, Rg3, Rg5, Rg6, Rh1, Rh2, Rh3, Rh4, Rk1 in active substance (powdered roots).

## Appendix C. Efficacy Outcome Measures

### Appendix C.1. Primary Outcome Measure

The d2 test is an internationally used and validated instrument that assesses both selective and sustained attention in a variety of clinical settings. It is a reliable and valid measure of attention based on German and United States normative samples, and it is a neuropsychological measure of selective and sustained attention, and visual-scanning speed. It is a paper-and-pencil test that asks participants to cross out any letter “d” with two marks above or below it in any order. The surrounding distractors are usually similar to the target stimulus, for example a “p” with two marks or a “d” with one or three marks. The d2 test form consists of 14 lines, each comprising 47 items for a total of 658 items. The subject is allowed 20 s per line, for a total of 4 min 40 s per test.

Outcome score: concentration-performance (CP) score, CP = TN − E2, where

○ TN score: total number of items processed for 4 min 40 s;
○ E1 score: omission errors—failure to respond to target;
○ E2 score: commission errors—errors made when responses given to nontargets;
○ E = E1 + E2: total errors;
○ E%: error percentage—allows for the interpretation of performance accuracy and carefulness; and
○ TP: total performance = TN − E—measures overall test performance.

### Appendix C.2. Secondary Outcome Measures

The Computerized Memory Flow Level 2 (CMFL2) test assesses cognitive functions (learning, memory, and attention). This test is available as an iPhone application (Mind Games, Brain Training Games by Mindware Consulting, Inc.—https://itunes.apple.com/se/app/mind-games-brain-training-games/id860325400?l=en&mt=8). The CMFL2 test was used in our study as a secondary efficacy endpoint to assess verbal memory, and provided the so-called accuracy score, which is the ratio of correct responses to total number of responses during 2 min expressed in a percentage scale from 0 to 100 (%). This test, based on the concept of continuous memory tasks, is valid for memory assessment since the relative standard deviation of this test is <5%, which is acceptable for quantitative analytical methods that provide reproducible data.

The PSS is the only empirically established index of general stress appraisal. It measures the degree to which situations in one’s life are appraised as stressful, and it is the most widely used validated psychological instrument for measuring the perception of stress. The scale also includes several direct queries about current levels of experienced stress. Questions in the PSS ask about feelings and thoughts during the last month. In each case, respondents are asked how often they feel a certain way. The PSS is a 10-item self-report questionnaire that measures a person’s evaluation of the stressfulness of situations in the past month of their lives [63]. Each 10-item report comprises five scores. The total score is the sum of 10 scores. Scores range from 0 to 50, with higher scores indicating greater stress. Items were designed to determine how unpredictable, uncontrollable, and overloaded respondents find their lives. Items 1–3, 6, 9, and 10 are negatively worded items; PSS-10 scores for these items are obtained by reversing the scores on the four positive items (e.g., 0 = 4, 1 = 3, 2 = 2, etc.), and then summing across all 10 items. Items 4, 5, 7, and 8 are positively worded items. For evaluation of the affectivity of HRG80, we compared the average of scores in each questionnaire.

Box A1. PSS 10-item inventory [62,63].
**Perceived Stress Scale**
The questions in this scale ask you about your feelings and thoughts **during the last month**. In each case, you will be asked to indicate by circling *how often* you felt or thought a certain way. Name ____________________________________________________________ Date _________ Age ________                    Gender (*Circle*): **M F** Other 
**0 = Never   1 = Almost Never    2 = Sometimes 3 = Fairly Often    4 = Very Often**
1. In the last month, how often have you been upset because of something that happened unexpectedly?..................................... **0 1 2 3 4** 2. In the last month, how often have you felt that you were unable to control the important things in your life?...................................................... **0 1 2 3 4** 3. In the last month, how often have you felt nervous and “stressed”? ........... **0 1 2 3 4** 4. In the last month, how often have you felt confident about your ability to handle your personal problems?................................................................. **0 1 2 3 4** 5. In the last month, how often have you felt that things were going your way?...................................................................................... **0 1 2 3 4** 6. In the last month, how often have you found that you could not cope with all the things that you had to do? ............................................................. **0 1 2 3 4** 7. In the last month, how often have you been able to control irritations in your life?....................................................................... **0 1 2 3 4** 8. In the last month, how often have you felt that you were on top of things?  **0 1 2 3 4** 9. In the last month, how often have you been angered because of things that were outside of your control? ....................................... **0 1 2 3 4** 10. In the last month, how often have you felt difficulties were piling up so high that you could not overcome them?............................... **0 1 2 3 4**

**Age**	**N**	**Mean**	**S.D.**
18–29	645	14.2	6.2
30–44	750	13.0	6.2
45–54	285	12.6	6.1
55–64	282	11.9	6.9
65 and older	296	12.0	6.3

### Appendix C.3. Safety Measures

Safety-outcome measures included incidence and severity of adverse events (AEs), and serious adverse events assessed by their nature and incidence according to Common Toxicity Criteria, Version 4.0. The subjects were informed to report any AE occurring during the study to the PI or the PI’s personnel. Open-ended standardized AE questions, such as “Have you had any health problems since you were last questioned?”, were asked by the PI (or designee) during each contact with the subject. This standardized questioning was done discretely to prevent the subjects from influencing each other. Any adverse events observed or reported by a subject and/or staff member was recorded in the CRF. Any AE including clinical findings not resolved on the final scheduled protocol day were followed up until they were resolved or explained. The following variables were recorded for each AE: event description, onset (date and time), resolution (date and time), maximal intensity, action taken, outcome, causality (yes or no), and whether or not it constituted a serious adverse event.

The intensity rating was defined as:

0 = mild (awareness of signs or symptoms, but easily tolerated),
0 = moderate (sufficient discomfort to interfere with normal activities), and
0 = severe (incapacitating, with inability to perform normal activities).

Safety-outcome measures were assessed from the date of randomization until the end of the treatment.

## Appendix E. CONSORT Checklist

**Table A2:** CONSORT checklist.

Section/Topic	Item No.	Checklist Item	Reported on Page No.
**Title and abstract**			
	1a	Identification as a randomized trial in the title.	1
	1b	Structured summary of trial design, methods, results, and conclusions (for specific guidance, see CONSORT for abstracts).	1
**Introduction**			
Background and objectives.	2a	Scientific background and explanation of rationale	2
	2b	Specific objectives or hypotheses.	2,8
**Methods**			
Trial design.	3a	Description of trial design (such as parallel, factorial), including allocation ratio.	9, Appendix A, Table 1
	3b	Important changes to methods after trial commencement (such as eligibility criteria), with reasons.	n/a
Participants.	4a	Eligibility criteria for participants.	9
	4b	Settings and locations where the data were collected.	8.9
Interventions.	5	Interventions for each group with sufficient details to allow replication, including how and when they were actually administered.	9,10, Appendix B
Outcomes.	6a	Completely defined prespecified primary and secondary outcome measures, including how and when they were assessed.	12,13, Table 1, Appendix C
	6b	Any changes to trial outcomes after the trial commenced, with reasons.	N/a
Sample size.	7a	How sample size was determined.	14
	7b	When applicable, explanation of any interim analyses and stopping guidelines.	N/a
Randomization:			
Sequence generation.	8a	Method used to generate random-allocation sequence.	10
	8b	Type of randomization, details of any restriction (such as blocking and block size).	10
Allocation-concealment mechanism.	9	Mechanism used to implement random-allocation sequence (such as sequentially numbered containers), describing any steps taken to conceal the sequence until interventions were assigned.	10
Implementation.	10	Who generated the random-allocation sequence, who enrolled participants, and who assigned participants to interventions.	10
Blinding.	11a	If done, who was blinded after assignment to interventions (for example, participants, care providers, those assessing outcomes) and how.	10,11
	11b	If relevant, description of intervention similarity.	10
Statistical methods.	12a	Statistical methods used to compare groups for primary and secondary outcomes.	13
	12b	Methods for additional analyses, such as subgroup and adjusted analyses.	Appendix D
**Results**			
Participant flow (diagram is strongly recommended).	13a	For each group, numbers of participants who were randomly assigned, received intended treatment, and were analyzed for the primary outcome.	3, Figure 1, Figure 2 and Figure 3, Table 1
	13b	For each group, losses and exclusions after randomization, with reasons.	3
Recruitment.	14a	Dates defining periods of recruitment and follow-up.	N
	14b	Why the trial ended or was stopped.	/a
Baseline data.	15	Table showing baseline demographic and clinical characteristics for each group.	Table 1
Analyzed numbers.	16	For each group, number of participants (denominator) included in each analysis, and whether analysis was by originally assigned groups.	3, Figure 1, Figure 2 and Figure 3, Table 1
Outcomes and estimation.	17a	For each primary and secondary outcome, results for each group, and the estimated effect size and its precision (such as 95% confidence interval)	Figure 1, Figure 2 and Figure 3, Appendix D
	17b	For binary outcomes, presentation of both absolute and relative effect sizes is recommended.	Figure 1, Figure 2 and Figure 3, Appendix D
Ancillary analyses.	18	Results of any other performed analyses, including subgroup and adjusted analyses, distinguishing prespecified from exploratory.	N/a
Harm.	19	All-important harmful or unintended effects in each group.	6
**Discussion**			
Limitations.	20	Trial limitations addressing sources of potential bias, imprecision, and, if relevant, multiplicity of analyses.	8
Generalizability.	21	Generalizability (external validity, applicability) of trial findings.	8
Interpretation.	22	Interpretation consistent with results, balancing benefits and harmful effects, and considering other relevant evidence.	8
**Other information**			
Registration.	23	Registration number and name of trial registry.	6
Protocol.	24	Where full trial protocol can be accessed, if available.	14
Funding.	25	Sources of funding and other support (such as supply of drugs), role of funders.	14
